# Selected Eye Symptoms

**Published:** 1894-04

**Authors:** Mahlon Preston

**Affiliations:** Norristown, Pa.


### SELECTED EYE SYMPTOMS.

MAHLON PRESTON, M. D., NORRISTOWN, PA.

The following indications for the treatment of ulcers on the cornea were published in THE HOMŒOPATHIC PHYSICIAN in September, 1886 (Vol. VI, page 341). To them I have added several other symptoms of considerable value.

### CORNEAL ULCERS.

*Central.* Ars., Cimicif., Euphra., Hepar, Merc., Nux-v., Sulph., (deep) Silic., Lac-felin.

*In upper part.* Crot-tig., Hepar (also serpiginous), Merc. (vascular), Rhus (vascular).

*At margin.* Hepar, Kali-b., Merc-iod., Thu. (pustule on edge of cornea, Rhus).

*Lower margin.* Hepar (vascular).

*Lower part.* Merc. (vascular superficial).

*Outer side.* Ars. (elevated edges).

*Outer margin.* (Sulph.)

*Inner part.* (Alumina.)

*Ulcer vascular.* Ars., Calc., Graph., Hepar, Merc., Rhus.

*Superficial.* Ars., Asaf., Canth., Con., Euphr., Merc-iod., Merc., Rhus, Sulph.

*Deep.* Hepar, Silicea, Sulph., Arg-n.

*Sloughing.* Hepar, Sil.

*Elevated.* Hepar.

*Red.* Hepar.

*Circular.* Hepar.

*Smooth.* Hepar.

*Serpiginous.* Hepar, Merc-iod.

*With white base.* Hepar.

*Perforating.* Sil., Podo.

*Elevated edges.* Ars.

*Non-vascular.* Sil.

*Transparent with clear edges.* Euphr.

(HOMŒOPATHIC PHYSICIAN, Vol. VI, page 341.)

*Ulcers right to l. on cornea, pustules on cornea.* Con-mac.

*Pteregium on eye with network of vessels.* Ars-met.

*Drawing through the right eye, mistiness of sight.* Paris-quad. (*Sympt. Codex*).

*Pain in the eye as if pulled into the head.* Paris-quad. (*Guiding Symptoms*).

*Eyes feel projecting with sensation as if a thread were drawn tightly through the ball and backward into the middle of the brain.* Paris-quad. (*Guiding Symptoms*).

*Some stitches through the middle of the eyes and some cloudiness before it.* Paris-quad. (Allen).

*From eyes to occiput.* Agar., Ang., Cuprum, Natr-s., Ran-bulb., Bov., Brom., Carb-v., Calc-caust., Cic., Cinnab., Colch., Coccus., Graph., Lith-c., Lyc., Lach., Phytolac., Sang., Spig., Tab., Thuj.

*Ulcerated (eye) sharp pains through into head.* Actea-rac.

*Darting into left eye as of needles run into cornea, worse on closing.* Actea-rac.

*As if eyeball were pulled toward temple by a thread.* Crotal-casc.

*From eye to head.* Acon., Agar., Ars., Badiag., Baryt., Bell., Berb., Bovist., Brom., Carb-v., Calc., Caust., Cicut., Cinnabar., Colch., Coccus-c., Graph., Lith-c., Lach., Phytol., Sang., Spigel., Tabacum, Thuja.

*From eye to occiput.* Cicuta., Cimicifuga, Coccus, Colch., Comoclad, Tabacum, Thuja.

*From eye to forehead.* Acon., Agar., Badiag., Bell., Berb. Brom., Calc., Caust., Lycop., Sang-c.

*From eye to temple.* Acon., Badiag., Baryt-c., Brom., Cinnabar., Coccus-c., Lith-c.

*From eye to vertex. Phytol-d. to top of head.* Cimicifuga-r. (darting).

